# HDAC10 inhibition represses melanoma cell growth and BRAF inhibitor resistance via upregulating SPARC expression

**DOI:** 10.1093/narcan/zcae018

**Published:** 2024-04-22

**Authors:** Hongbo Ling, Yixuan Li, Changmin Peng, Shengyu Yang, Edward Seto

**Affiliations:** George Washington Cancer Center, Department of Biochemistry & Molecular Medicine, The George Washington University School of Medicine & Health Sciences, Washington, DC 20037, USA; George Washington Cancer Center, Department of Biochemistry & Molecular Medicine, The George Washington University School of Medicine & Health Sciences, Washington, DC 20037, USA; George Washington Cancer Center, Department of Biochemistry & Molecular Medicine, The George Washington University School of Medicine & Health Sciences, Washington, DC 20037, USA; Department of Cellular and Molecular Physiology, Penn State Cancer Institute, The Penn State University, 400 University Drive, Hershey, PA 17033, USA; George Washington Cancer Center, Department of Biochemistry & Molecular Medicine, The George Washington University School of Medicine & Health Sciences, Washington, DC 20037, USA

## Abstract

Secreted protein acidic and rich in cysteine (SPARC), a conserved secreted glycoprotein, plays crucial roles in regulating various biological processes. SPARC is highly expressed and has profound implications in several cancer types, including melanoma. Understanding the mechanisms that govern SPARC expression in cancers has the potential to lead to improved cancer diagnosis, prognosis, treatment strategies, and patient outcomes. Here, we demonstrate that histone deacetylase 10 (HDAC10) is a key regulator of SPARC expression in melanoma cells. Depletion or inhibition of HDAC10 upregulates SPARC expression, whereas overexpression of HDAC10 downregulates it. Mechanistically, HDAC10 coordinates with histone acetyltransferase p300 to modulate the state of acetylation of histone H3 at lysine 27 (H3K27ac) at *SPARC* regulatory elements and the recruitment of bromodomain-containing protein 4 (BRD4) to these regions, thereby fine-tuning *SPARC* transcription. HDAC10 depletion and resultant SPARC upregulation repress melanoma cell growth primarily by activating AMPK signaling and inducing autophagy. Moreover, SPARC upregulation due to HDAC10 depletion partly accounts for the resensitization of resistant cells to a BRAF inhibitor. Our work reveals the role of HDAC10 in gene regulation through indirect histone modification and suggests a potential therapeutic strategy for melanoma or other cancers by targeting HDAC10 and SPARC.

## Graphical Abstract

**Graphical Abstract:**
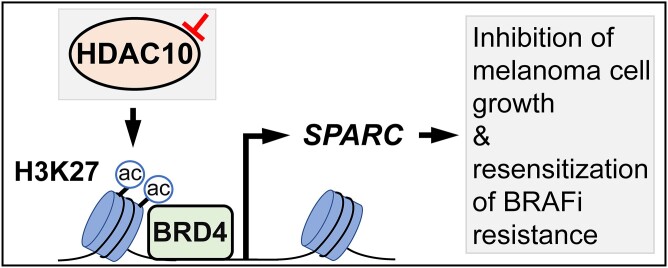

## Introduction

Secreted protein acidic and rich in cysteine (SPARC), also known as osteonectin (ON) or basement-membrane protein 40 (BM-40), is a highly conserved glycoprotein present across invertebrates and vertebrates. First reported in bone tissues, SPARC possesses the ability to interact with collagen fibrils and hydroxyapatite at discrete locations, underscoring its pivotal role in the process of bone mineralization (1). Later studies identified the presence of SPARC in endothelial cells and a diverse array of other tissues (2,3), and characterized it as an extracellular matrix (ECM)-associated protein that is vital for tissue remodeling (4), morphogenesis (5,6), and angiogenesis (7,8). Over the years, the comprehensive study of SPARC has revealed that it is an integral regulator in a myriad of physiological and pathological processes, including tumorigenesis (9–13).

Depending on the specific cell types and animal models under study, SPARC can manifest contrasting roles in cancer (13). This can be attributed to variations in its expression, secretion, and wide-ranging biological functions. Produced and secreted by various cell types, spanning from cancer cells to stromal and immune cells, SPARC influences the tumor microenvironment in several ways. For example, SPARC plays instrumental roles in the deposition, assembly, and remodeling of ECM, encompassing processes like collagen transformation and matrix protein secretion (14–16). Further, by interactions with soluble factors and consequent alteration in signaling pathways, SPARC impacts proliferation and migration in cancer cells (7,17–19). In addition, SPARC can alter the tumor microenvironment by dampening immune cell infiltration (20,21). While it is most often encountered as a secreted glycoprotein, SPARC is also localized on the cell surface and within the intracellular compartment in several cell types. Within the cell, particularly when associated with membranes, SPARC plays a role in pivotal cellular signaling pathways, impacting cell proliferation, differentiation, and survival (22,23). Its interactions with cytoskeletal elements, such as actin and tubulin, underline its role in the regulation of migration and structural organization (24–26).

Despite SPARC’s multifaceted roles across various cancer types, it's undeniable that modifying its expression and activity can significantly alter tumor and cellular characteristics. For instance, SPARC inhibits proliferation in ovarian cancer (27,28), breast cancer (29), and medulloblastoma (30). Furthermore, it mitigates peritoneal carcinomatosis and metastasis in these cancers (31). Consistently, exogenous SPARC administration has demonstrated a suppression of growth in a spectrum of cancers including pancreatic cancer (32), colorectal cancer (33), neuroblastomas (34), and leukemia (35). SPARC has also been implicated in chemoresistance. In the realm of colorectal cancer, SPARC enhances the chemosensitivity of resistant cells either paired with chemotherapy agents (33) or combined with vitamin D (36). Similarly, high SPARC expression is associated with better outcomes of non-small cell lung cancer patients treated with cisplatin (37). Accordantly, highly expressed and exogenous SPARC displayed an increased susceptibility of melanomas to the growth-inhibitory impact of drugs like cisplatin (38). These collective findings bolster the idea of targeting SPARC therapeutically in various malignancies, and a deeper exploration into the molecular mechanisms that regulate SPARC expression in cancers is imperative.

Histone deacetylases (HDACs) are enzymes that catalyze the removal of acetyl groups from ϵ-N-acetyl lysine residues on histone and non-histone proteins (39,40). These enzymes are involved in various key cellular processes, including but not limited to cell cycle progression, apoptosis, and differentiation. Aberrant HDAC function has been implicated in the pathogenesis of numerous human diseases, particularly in cancer. In light of this, several HDAC inhibitors (HDACi) have been approved for clinical use in certain cancer types (41–43). In humans, 18 HDAC enzymes have been identified and are grouped into four classes based on their similarity to yeast homologs. Class I consists of HDAC1−3, and 8, which are highly homologous to the yeast HDAC Rpd3. Class II comprises HDAC4−7, 9 and 10, with their deacetylase domains closely related to that of yeast Hda1. Class III includes seven members of the sirtuin proteins (SIRT1−7) that exhibit homology to the silent information regulator 2 (Sir2). HDAC11 is the sole member of class IV. While classes I, II and IV are zinc-dependent enzymes showing sequence similarity to each other, they lack homology with Sir2-related proteins that require NAD^+^. The reversible acetylation and deacetylation of histones play a critical role in gene expression regulation. Generally speaking, when histone acetyltransferases (HATs) acetylate histones, the resultant neutralization of the lysine residues diminishes histone-DNA affinity, leading to chromatin loosening and subsequent gene transcription. In opposition, deacetylation by HDACs enhances histone-DNA interaction, compacting the DNA and repressing transcription (44). HDACs and HATs also modulate the acetylation state of numerous non-histone proteins, which impacts the protein's structure, function or interactions, often altering gene transcription (39,45). In addition, HDACs might not change the acetylation state of histone and non-histone protein partners. Instead, they may regulate gene transcription by directly interacting with and, in turn, affecting the function of transcription (co)factors (46). In essence, while HDACs are known for their diverse gene regulatory roles and implications in both cancer and non-cancerous diseases, their specific influence on SPARC expression, particularly in the cancer landscape, remains unknown.

In this study, we demonstrate that HDAC10 is pivotal in orchestrating SPARC expression. HDAC10 depletion or inhibition resulted in an elevated level of acetylation of histone H3 at lysine 27 (H3K27ac) and augmented recruitment of bromodomain-containing protein 4 (BRD4) at *SPARC* regulatory elements, thereby promoting *SPARC* transcription. Furthermore, our work shows that HDAC10 depletion and resultant SPARC upregulation led to repressive melanoma cell growth and increased vulnerability to BRAF inhibition in resistant cells. These findings uncover a novel mechanism by which HDAC10 regulates gene expression through histone modification and suggest that targeting HDAC10 and SPARC may hold therapeutic potential in combating melanoma and other malignancies.

## Materials and methods

### Cell lines

The following cell lines were used in this study: HEK293T (ATCC, CRL-3216, RRID:CVCL_0063), A375 (ATCC, CRL-1619, RRID:CVCL_0132), SK-MEL-2 (ATCC, HTB-68, RRID:CVCL_0069), SK-MEL-5 (ATCC, HTB-70, RRID:CVCL_0527), SK-MEL-28 (ATCC, HTB-72, RRID:CVCL_0526), HMEL-BRAF^V600E^ (RRID:CVCL_0132), UACC257 (RRID:CVCL_1779), WM983A (RRID:CVCL_6808), WM3918 (RRID:CVCL_0526), 1205Lu (RRID:CVCL_5239), WM793 (RRID:CVCL_8787), A549 (ATCC, CCL-185, RRID:CVCL_0023), H1299 (ATCC, CRL-5803, RRID:CVCL_0060) and wild-type (WT) and *Hdac10*-knockout (KO) mouse embryonic fibroblasts (MEFs) (47). Cells were grown in Dulbecco's modified Eagle's medium (DMEM) supplemented with 10% fetal bovine serum (FBS) and 1% penicillin–streptomycin at 37°C in a humidified atmosphere of 5% CO_2_.

### Antibodies

The antibodies used in this study are: anti-HDAC10 (Sigma-Aldrich, #H3413, RRID:AB_261940), anti-FLAG epitope (M2; Sigma-Aldrich, #F1804, RRID:AB_262044), anti-α-tubulin (12G10; Developmental Studies Hybridoma Bank; RRID:AB_2315509), and anti-acetyl-tubulin (clone 6-11B-1; Sigma-Aldrich, #T7451, RRID:AB_609894), anti-β-actin (Proteintech, #66009-1-IG, RRID:AB_2687938), anti-V5 epitope (Thermo Fisher Scientific, #MA5-15253, RRID:AB_10977225), anti-SPARC (Cell Signaling Technology, #5420, RRID:AB_10692794), anti-acetyl-Histone H3 (Lys27) (Cell Signaling Technology, #4353, RRID:AB_10545273), anti-Histone H3 (Cell Signaling Technology, #9715, RRID:AB_331563), anti-BRD4 (Cell Signaling Technology, #13440, RRID:AB_2687578), anti-phospho-AMPK (T172) (Cell Signaling Technology, #2531, RRID:AB_330330), anti-AMPK (Cell Signaling Technology, #2532, RRID:AB_330331), anti-phospho-acetyl-CoA carboxylase (Ser79) (Cell Signaling Technology, #3661, RRID:AB_330337), anti-acetyl-CoA carboxylase (Cell Signaling Technology, #3662, RRID:AB_2219400), anti-phospho-Tuberin/TSC2 (Ser1387) (Cell Signaling Technology, #5584, RRID:AB_10698883), anti-Tuberin/TSC2 (Cell Signaling Technology, #4308, RRID:AB_10547134), anti-phospho-p70 S6 Kinase (Thr389) (Cell Signaling Technology, #9234, RRID:AB_2269803), anti-p70 S6 Kinase (Cell Signaling Technology, #2708, RRID:AB_390722), anti-phospho-ERK1/2 (Thr202/Tyr204) (Cell Signaling Technology, #9101, RRID:AB_331646), anti-ERK1/2 (Cell Signaling Technology, #9102, RRID:AB_330744), anti-phospho-AKT (Ser473) (Cell Signaling Technology, #4060, RRID:AB_2315049), anti-phospho-AKT (Ser473) (Cell Signaling Technology, #9272, RRID:AB_329827), anti-phospho-c-JUN (Ser63) (Cell Signaling Technology, #2361, RRID:AB_490908), anti-c-JUN (Cell Signaling Technology, #9165, RRID:AB_2130165), anti-phospho-SMAD2 (Ser465/Ser467) (Cell Signaling Technology, #18338, RRID:AB_2798798), anti-SMAD2 (Cell Signaling Technology, #5339, RRID:AB_10626777), anti-phospho-SMAD3 (Ser423/425) (Cell Signaling Technology, #9520, RRID:AB_2193207), anti-SMAD3 (Cell Signaling Technology, #9523, RRID:AB_2193182), anti-Beclin-1 (Cell Signaling Technology, #3495, RRID:AB_1903911), anti-p62 (Cell Signaling Technology, #5114, RRID:AB_10624872), anti-LC3A/B (Cell Signaling Technology, #12741, RRID:AB_2617131), anti-PARP (Cell Signaling Technology, #9542, RRID:AB_2160739), anti-cleaved Caspase 3 (Cell Signaling Technology, #9661, RRID:AB_2341188), anti-cyclin D1 (Santa Cruz Biotechnology, #sc-753, RRID:AB_2070433), anti-cyclin E (Santa Cruz Biotechnology, #sc-198, RRID:AB_631346), anti-cyclin A (Santa Cruz Biotechnology, #sc-751, RRID:AB_631329), anti-cyclin B1 (Santa Cruz Biotechnology, #sc-752, RRID:AB_2072134), anti-p21 (Santa Cruz Biotechnology, #sc-397, RRID:AB_632126), anti-p27 (Santa Cruz Biotechnology, #sc-527, RRID:AB_632131), anti-p300 (Santa Cruz Biotechnology, #sc-585, RRID:AB_2231120), anti-vinculin (Santa Cruz Biotechnology, #sc-73614, RRID:AB_1131294), anti-Caspase 3 (BD Biosciences, #610322, RRID:AB_397713), anti-acetyl-Histone H4 (pan-acetyl) (Active Motif, #39244, RRID:AB_2793201) and anti-acetyl-Histone H3 (pan-acetyl) (Active Motif, #39140, RRID:AB_2687871).

### Chemical reagents

Trichostatin A (#T8552), Bufexamac (#B0760), Tubacin (#SML0065), MS-275 (#EPS002), and sodium butyrate (#B5887) were obtained from Sigma-Aldrich. DKFZ-748 (#CS-0617374) was purchased from Chemscene. Vemurafenib (PLX4032, #RG7204) was purchased from Selleck Chemicals. A-485 (#24119) and Bafilomycin A1 (#11038) were from Cayman Chemical Company.

### Plasmids and lentiviruses

The lentiviral vector pLenti-CMV-Neo-DEST (Addgene, #17392, RRID:Addgene_17392) was a gift from Eric Campeau & Paul Kaufman (48). The pLenti-CMV-Neo-DEST-HDAC10 was described previously (47). The plasmid lentiCRISPR v2 (Addgene, #52961, RRID:Addgene_52961) was a gift from Feng Zhang (49). *HDAC10* CRISPR/Cas9 knockout plasmid was generated by inserting guide RNA (gRNA) sequences targeting 5′-GACGCTCGATCTCGCACTCGGGG-3′ into lentiCRISPR v2. The pLKO.1-based lentiviral plasmids for delivering short hairpin RNAs (shRNAs) targeting human HDAC1−11, SPARC, p300 and BRD4 were from Sigma-Aldrich. The plasmid pLKO.1 GFP shRNA (Addgene, #30323, RRID:Addgene_30323) was a gift from David Sabatini (50).The target sequences of shRNAs are described in Supplementary Table S1. The plasmids pcDNA-dCas9-p300 Core (Addgene, #61357, RRID:Addgene_61357) and pcDNA-dCas9-p300 Core (D1399Y) (Addgene, #61358, RRID:Addgene_61358) were gifts from Charles Gersbach (51). The plasmid pCDNA-dCas9-HDAC10 was generated by replacing the p300 Core encoding sequence of plasmid pcDNA-dCas9-p300 Core with HDAC10 cDNA. The plasmid lenti-sgRNA blast (Addgene, #104993, RRID:Addgene_104993), a gift from Brett Stringer (52), was used to deliver gRNAs targeting *SPARC* locus, as listed in Supplementary Table S1. The plasmid pLX304-SPARC (#HsCD00444770) was purchased from DNASU.

Lentiviruses were prepared by transfecting HEK293T cells with the lentiviral expression plasmids together with the lentiviral packaging plasmids (pLP-1, pLP-2 and pLP/VSVG) (Invitrogen). Lentivirus-transduced cells were selected for neomycin (G418) or puromycin resistance.

### *In vivo* tumor growth

For assessment of the effect of HDAC10 on melanoma growth, HDAC10 knockdown and control A375 cells (1 × 10^6^ in 100 μl PBS) were subcutaneously injected into the flanks of NSG mice at the age of 6–8 weeks (Jackson Laboratory, RRID:IMSR_JAX:005557). The mice were sacrificed 3 weeks after injection and the tumor weight was measured. For the investigation of lung and liver colonization by melanoma cells, cells were injection into NSG mice via tail vein. Mice were euthanized 4–5 weeks post-injection. The influence of HDAC10 on lung and liver colonization of melanoma cells was assessed by measuring body weight, lung weight, calculating the ratio of lung weight to body weight, and counting the number of surface tumors. This was complemented by a subsequent histopathological analysis. All animal experiments were conducted in accordance with the ethical guidelines and approved by the Institutional Animal Care and Use Committee of The George Washington University. Protocols #A436, #A2023-003 and #A2023-016 are specifically applicable to the experiments reported in this paper.

### Real-time quantitative PCR (RT-qPCR)

Total RNA was extracted from cells with the mirVana miRNA isolation kit (Ambion, #AM1560) or TRIzol Reagent (Thermo Fisher Scientific, #15596018), and was reverse transcribed into cDNA using the qScript cDNA synthesis kit (Quanta Biosciences, #95047). Relative quantitation of mRNAs was carried out via SYBR green-based quantitative PCR using a Quantstudio 3 Real-time PCR system (Applied Biosystems) with primers shown in Supplementary Table S1. PCR results were analyzed using the 2^−(ΔΔCT)^ method.

### Colony formation assay

Cells were seeded on 3.5-cm dishes or 6-well plates. After appropriate periods, cells were washed with PBS and stained with 1% crystal violet for 30 min at room temperature. The resultant colonies were subsequently counted, imaged, and the relative growth was quantified using ImageJ software (RRID:SCR_003070).

### Spheroid growth assay

Three-dimensional spheroid culture was done as described previously (53). Briefly, 96-well tissue culture plates were pre-coated with 75 μl of 1% agarose in PBS. Nearly confluent cells were trypsinized and seeded at 10^4^ cells/well in 150 μl of DMEM to obtain a single homotypic spheroid per well. Every 3 days, 75 μl of supernatant was delicately removed from each well and replenished with fresh medium. On day 6, the spheroid size was evaluated using an inverted microscope. Spheroid volume was determined using the equation *V* = (*LW*^2^) × 0.5, where *L* is length and *W* is width of the spheroid.

### Immunoblotting

Protein samples (cell lysates or media) were resolved by SDS-PAGE, and electrotransferred onto a nitrocellulose membrane. The blots were incubated in blocking buffer [5% (w/v) nonfat dry milk in TBS + 0.05% Tween 20 (TBS-T)] for 1 h, and incubated with primary antibodies for 16 h at 4°C, followed by incubation with horseradish peroxidase (HRP)-linked goat anti-rabbit IgG (Cell Signaling Technology, #7074, RRID:AB_2099233) or HRP-linked goat anti-mouse IgG (Cell Signaling Technology, #7076, RRID:AB_330924) for 1 h at room temperature. The antibody-antigen complex was visualized by ECL chemiluminescence.

### Indirect immunofluorescence

Cells were fixed and permeabilized. Cells were blocked by 10% normal goat serum (Sigma-Aldrich, #G9023) in PBS for 1 h, then were incubated with primary antibodies for 1 h and with either Alexa Fluor™ 594-conjugated goat anti-mouse IgG (H + L; Invitrogen, #A11005, RRID:AB_2534073) or Alexa Fluor™ 488-conjugated goat anti-rabbit IgG (H + L; Invitrogen, #A11008, RRID:AB_143165) for 1 h. All immunostaining experiments in this study were accompanied with DAPI staining. The BrdU-incorporation assay was performed using the BrdU Labeling and Detection Kit I (Roche, #11296736001, RRID:AB_2814711) according to the manufacturer's instruction.

### Chromatin immunoprecipitation (ChIP) assay

ChIP assays were performed essentially as described previously (47). Briefly, cells were incubated for 10 min in 1% formaldehyde, followed by addition of 0.125 M glycine and sonication to generate DNA fragments of 200–1000 bp. Chromatin from about 1.5 × 10^6^ cells was incubated overnight at 4°C with 2–5 μg antibodies to FLAG epitope (M2; Sigma-Aldrich, #F1804, RRID:AB_262044), BRD4 (Cell Signaling Technology, #13440, RRID:AB_2687578), or acetyl-Histone H3 (Lys27) (Cell Signaling Technology, #4353, RRID:AB_10545273). Normal mouse IgG (Santa Cruz Biotechnology, #sc-2025, RRID:AB_737182) and normal rabbit IgG (Millipore, #12-370, RRID:AB_145841) were used as controls. Samples were incubated with preblocked protein A/G Plus beads (Santa Cruz Biotechnology, #2003, RRID:AB_10201400) for 2 h at 4°C. DNA was purified and then amplified by PCR using the primers shown in Supplementary Table S1.

### Statistical analysis

Statistical analyses were carried out using GraphPad Prism 8 (RRID:SCR_002798) or Microsoft Excel. The normality of continuous variables was analyzed by Shapiro-Wilk test. For normally distributed data, comparisons were made using the un-pooled two-sample *t*-test. Differences were considered statistically significant when the *P*-value was <0.05.

## Results

### HDAC10 represses SPARC expression in melanoma cells

**Figure 1.**
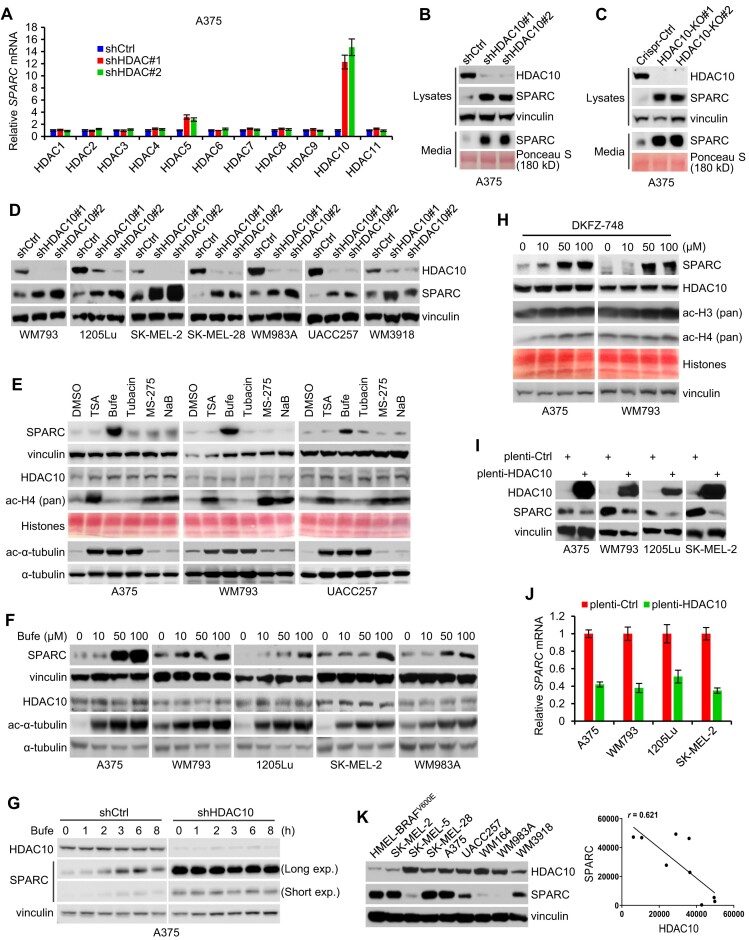
HDAC10 represses SPARC expression in melanoma cells. (**A**) A375 melanoma cells were infected with lentiviruses harboring shRNA targeting individual HDAC (HDAC1–11). Relative levels of *SPARC* mRNA of the cells were determined by qPCR. (**B**) A375 cells were infected with lentiviruses harboring HDAC10 or control shRNA. Cell lysates were immunoblotted with antibodies to HDAC10, SPARC, and vinculin (loading control). Additionally, culture media proteins were separated, stained with Ponceau S, and immunoblotted for SPARC. (**C**) A375 cells were deleted of HDAC10 by CRISPR/Cas9-mediated knockout. The SPARC expression of the cells was examined by immunoblotting. (**D**) HDAC10 was depleted and SPARC expression was examined across multiple melanoma cell lines. (**E**) Melanoma cells were exposed to 0.33 μM Trichostatin A (TSA), 0.1 mM Bufexamac (Bufe), 2 μM Tubacin, 1 μM MS-275, 5 mM sodium butyrate (NaB) or the control for 24 h, then were examined for the expression of SPARC and HDAC10. The levels of acetylated histone (ac-H4) and acetylated α-tubulin (ac-α-tubulin) were detected to show the inhibition efficacies of class I HDACs and HDAC6, respectively. (**F**) Melanoma cells were treated with Bufe or the control for 24 h, and SPARC expression was examined. (**G**) HDAC10-depleted and control A375 cells were treated with 0.1 mM Bufe for 1–8 h, then were examined for SPARC expression. (**H**) Cells were treated with HDAC10 inhibitor DKFZ-748 for 24 h, and were immunoblotted with indicated antibodies. (**I**) HDAC10 was forcedly expressed, and lysates were immunoblotted for SPARC. (**J**) The relative levels of *SPARC* mRNA of cells overexpressing HDAC10 or control were quantified. (**K**) Immunoblotting (left panel) and correlation (right panel) analyses of HDAC10 and SPARC protein levels in a panel of melanoma cell lines.

To explore the potential relationship of HDACs and SPARC, we performed knockdowns of individual classical HDACs (HDAC1−11) in A375 melanoma cells using lentivirus-delivered shRNA (Supplementary Figure S1). *SPARC* mRNA level was then evaluated. The knockdown of HDAC10 resulted in a marked increase in *SPARC* mRNA level, whereas the knockdown of HDAC5 resulted in a modest increase in *SPARC* mRNA. There were no significant *SPARC* mRNA changes from depletion of the other nine HDACs. Thus, HDAC10 is the major HDAC that suppresses SPARC expression in melanoma cells (Figure 1A). Consistently, knockdown (Figure 1B) or knockout (Figure 1C) of HDAC10 in A375 cells resulted in robustly elevated intracellular and extracellular accumulation of SPARC protein. Similar results were obtained when other melanoma cell lines were depleted of HDAC10 (Figure 1D). Complementary to these observations, treatment with the class IIb HDAC (HDAC10 and HDAC6) inhibitor Bufexamac (54) led to an augmented SPARC protein level; in contrast, exposure to either the broad spectrum HDAC inhibitor Trichostatin A, or the HDAC6 selective inhibitor Tubacin, or the class I HDACs inhibitors MS-275 and sodium butyrate, none of which efficiently targets HDAC10, has little effect on SPARC expression; suggesting that Bufexamac targets HDAC10, rather than HDAC6 or other HDACs, to regulate SPARC expression (Figure 1E). Bufexamac upregulated SPARC expression in a dose-dependent manner (Figure 1F). The most significant induction of SPARC was observed when Bufexamac was applied at 100 μM, despite HDAC6 activity being fully inhibited at a lower concentration (50μM), as evidenced by the saturated accumulation of acetylated tubulin, reinforcing an HDAC6-independent mechanism for SPARC regulation by Bufexamac. Corroboratively, when HDAC10 was depleted, the effect of Bufexamac on SPARC expression was largely blunted (Figure 1G). Concordantly, the employment of a recently developed HDAC10 selective inhibitor, DKFZ-748 (55), resulted in elevated SPARC expression (Figure 1H). Conversely, overexpression of HDAC10 reduced SPARC at both protein (Figure 1I) and mRNA (Figure 1J) levels. Thus, HDAC10 suppresses SPARC expression in melanoma cells. In support of this notion, the level of endogenous HDAC10 protein is statistically inversely correlated with endogenous SPARC protein expression in a panel of melanoma cell lines (Figure 1K).

### HDAC10 modulates the H3K27ac state at *SPARC* regulatory elements

**Figure 2.**
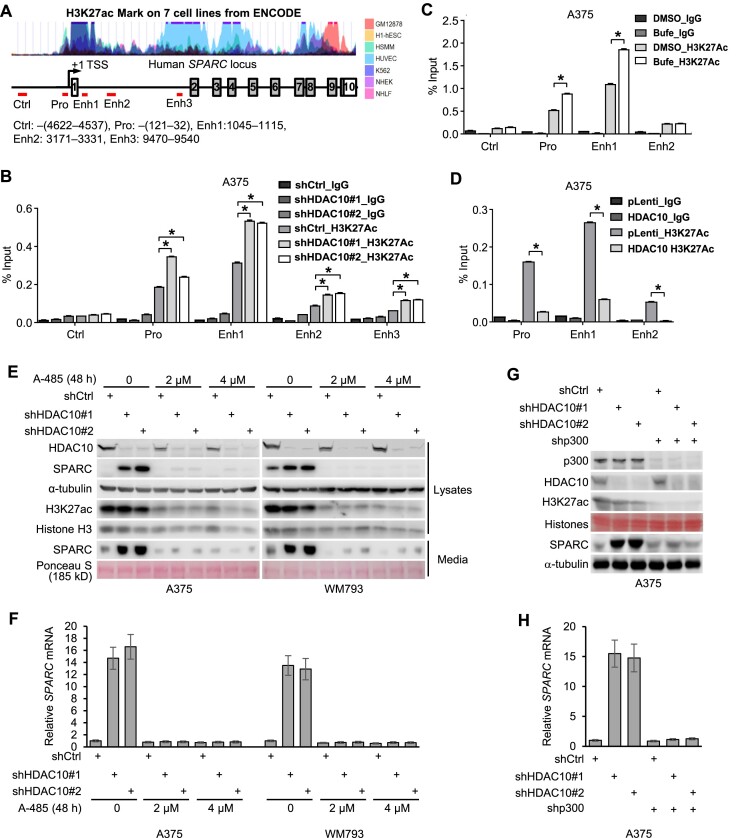
HDAC10 modulates the H3K27ac state at *SPARC* regulatory elements. (**A**) Schematic depiction of the chromatin modification pattern at *SPARC* locus on chromosome 5, adapted from the UCSC Genome Browser database. The +1 position represents the primary transcription initiation site. Red horizontal bars highlight regions amplified by qPCR post-ChIP. (**B**) ChIP analysis of H3K27ac level at *SPARC* locus in HDAC10-depleted and control A375 cells. *P < 0.05. (**C**) Cells were treated with Bufexamac (Bufe) or the control (DMSO). The H3K27ac level at *SPARC* regulatory elements was evaluated by ChIP. *P < 0.05. (**D**) A375 cells were overexpressed with HDAC10. The H3K27ac level at *SPARC* locus was assessed by ChIP assays. *P < 0.05. (E, F) HDAC10-depleted and control A375 and WM793 cells were treated with p300/CBP inhibitor A-485 (2 or 4 μM) for 48 h. Cell lysates were analyzed for intracellular levels of HDAC10, SPARC, H3K27ac, and Histone H3. Additionally, secreted SPARC protein from culture media was evaluated (**E**). *SPARC* mRNA levels were quantified using qPCR (**F**). (G, H) HDAC10-depleted or control A375 cells were further depleted of p300. The expression of SPARC protein (**G**) and mRNA (**H**) was determined.

Consistent with our results that HDAC10 represses SPARC expression in melanoma cells, in an RNA-seq analysis, *SPARC* is one of the top upregulated genes upon HDAC10 depletion in H1299 lung cancer cells (Supplementary Table S2; Supplementary Figure S2A). Notably, the basal *SPARC* expression varies across distinct cancer and/or cell types, e.g. the relative level of *SPARC* mRNA in melanoma cells (A375 and WM793) is ∼400–1000 times higher than in lung cancer cell lines (H1299 and A549) (Supplementary Figure S2B). HDAC10 depletion consistently led to a significant increase in the expression of *SPARC* mRNA, regardless of their basal levels (Supplementary Figure S2C). Furthermore, *Hdac10*-KO MEFs exhibited higher expression levels of SPARC, compared to the WT MEFs (Supplementary Figure S2D). Therefore, the transcriptional repression of *SPARC* by HDAC10 is not cell-type dependent. Alternatively, HDAC10 may target certain machinery that is commonly shared across cell lines, such as histones. Histones are known to possess multiple acetylation sites, including lysine residues on histones H3, H4, H2A, and H2B. Among these acetylation sites, H3K27ac, primarily catalyzed by p300/CBP acetyltransferase(s) (56), is a well-recognized marker of active gene regulatory elements crucial for the initiation and elongation of gene transcription. Interestingly, the promoter (Pro: –121 to –32) and enhancers (Enh1: 1045 to 1115; Enh2: 3171 to 3331; Enh3: 9470 to 9540) of *SPARC* are putatively acetylated on H3K27 according to the prediction of the ENCODE database (Figure 2A). To understand whether HDAC10 can regulate the H3K27ac state at these loci, we performed ChIP assays using an antibody to H3K27ac. Compared to a control region (–4622 to –4537), H3K27ac was significantly enriched at multiple *SPARC* promoter and enhancers when HDAC10 was depleted (Figure 2B) or pharmaceutically inhibited (Figure 2C). In contrast, ectopic HDAC10 expression reduced the H3K27ac level at these regions (Figure 2D). Collectively, our results suggest HDAC10 plays a role in modulating the H3K27ac state at *SPARC* regulatory elements.

Given the importance of H3K27ac in gene expression regulation, the observed increase in H3K27ac level may contribute to the SPARC upregulation by HDAC10 depletion. To corroborate this hypothesis, cells were depleted of HDAC10 and/or treated with A-485, a selective p300/CBP inhibitor (57). The H3K27ac level and the SPARC expression were then examined. Exposure to A-485 blocked the H3K27 acetylation and entirely abolished the SPARC upregulation (Figures 2E and F). Consistently, depletion of p300, which significantly reduced the overall level of H3K27ac in cells, attenuated the SPARC upregulation (Figures 2G and H); suggesting that HDAC10 regulates *SPARC* transcription, at least partially, by modulating the H3K27ac state through its interplay with p300/CBP.

### Bidirectional regulation of the H3K27ac state and BRD4 accumulation at *SPARC* regulatory elements by dCas9-HDAC10 and dCas9-p300

**Figure 3.**
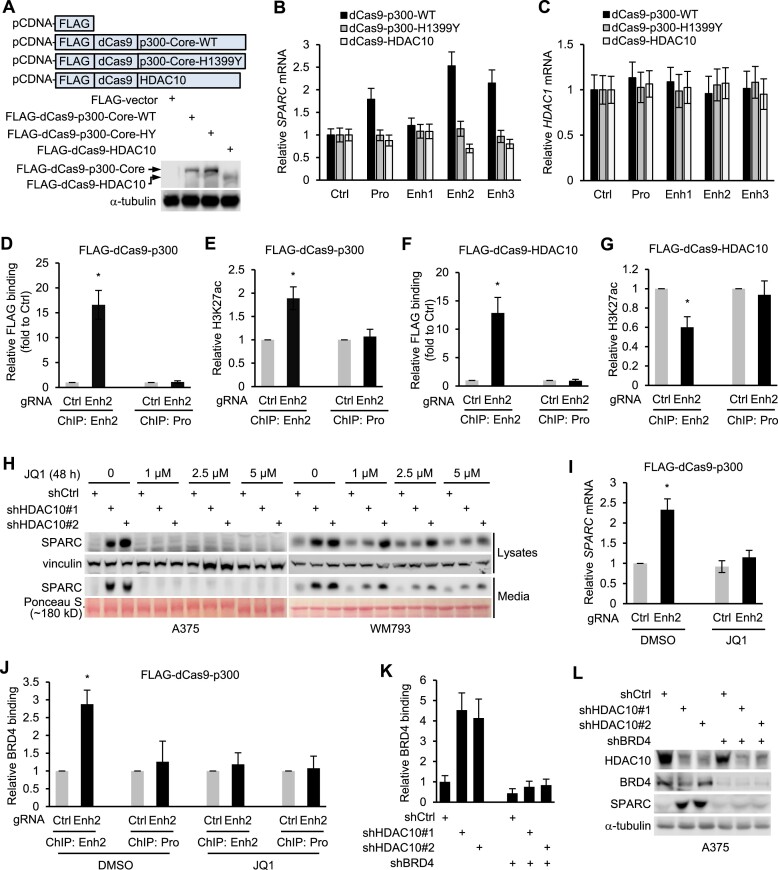
Bidirectional regulation of the H3K27ac state and BRD4 accumulation at *SPARC* regulatory elements by dCas9-HDAC10 and dCas9-p300. (**A**) A375 cells were transfected with plasmids expressing FLAG-tagged dCas9-fused p300 Core (WT or H1399Y) or HDAC10. Cell lysates were immunoblotted with antibodies to FLAG epitope and α-tubulin. (B, C) Cells were transfected with plasmids expressing dCas9 fused proteins and gRNAs targeting *SPARC* promoter, enhancers or control region, respectively. The relative mRNA levels of *SPARC* (**B**) and *HDAC1* (**C**) were quantified by qPCR. (D, E) Cells were transfected with plasmids expressing FLAG-tagged dCas9-fused p300 and gRNAs targeting Enh2. The relative accumulation of FLAG-tagged dCas9-fused p300 (**D**) and H3K27ac level (**E**) at Enh2 and the promoter (serves as control) were determined by ChIP assays with antibodies to FLAG epitope and H3K27ac. *P < 0.05. (F, G) Cells were transfected with plasmids expressing FLAG-tagged dCas9-fused HDAC10 and gRNAs targeting Enh2. The relative accumulation of FLAG-tagged dCas9-fused HDAC10 (**F**) and H3K27ac (**G**) at Enh2 and the promoter were determined by ChIP. *P < 0.05. (**H**) HDAC10-depleted and control cells were treated with JQ1 for 24 h. Cells were washed and maintained in fresh media containing JQ1 for an additional 24 h. Cell lysates and media were blotted for SPARC. (I, J) Cells were transfected with plasmids expressing FLAG-tagged dCas9-p300 and gRNAs to Enh2 and control region. Cells were then treated with JQ1 or control. The relative level of *SPARC* mRNA was quantified by qPCR (**I**). The relative BRD4 binding to Enh2 and control region were evaluated by ChIP with an antibody to BRD4 (**J**). *P < 0.05. (K, L) Cells were depleted of HDAC10 and/or BRD4. The relative BRD4 binding to Enh2 was determined by ChIP (**K**). Cell lysates were blotted with antibodies to indicated proteins (**L**).

It is important to note that HDAC10 depletion did not significantly alter the overall level of H3K27ac in cells (Figures 2E and G). HDAC10 might work in tandem with p300/CBP to fine-tune transcription by modulating local H3K27ac accumulation at *SPARC* locus. To validate this hypothesis, we employed a CRISPR-based approach to selectively tether catalytically inactive Cas9 (dCas9)-fused HDAC10, p300 Core WT or H1399Y (acetyltransferase inactive mutant) to the putative *SPARC* promoter or enhancers. The A375 cells, in which the SPARC expression and H3K27ac level are highly responsive to HDAC10 or p300/CBP modulation, were employed for these experiments. Cells were co-transfected with plasmids expressing dCas9-fused proteins and gRNAs precisely targeting *SPARC* promoter, enhancers, or control region. The cells transfected with the expression plasmids had a similar expression level of the dCas9-fused proteins (Figure 3A). When co-expressing with the promoter, Enh2 or Enh3 gRNAs, the dCas9-p300 and dCas9-HDAC10 led to a significant increase and decrease in *SPARC* mRNA level, respectively. Expression of dCas9-p300-H1399Y did not significantly change *SPARC* mRNA level, reaffirming the instrumental role of the H3K27ac state in modulating *SPARC* expression (Figure 3B). As a control, *HDAC1* expression remained consistent across all co-transfection conditions (Figure 3C), confirming that the alterations in *SPARC* expression can be attributed to the targeted engagement of dCas9-p300 or dCas9-HDAC10 with *SPARC* regulatory elements, as opposed to unintended off-target interactions.

Three of the four putative regulatory elements played a role in regulating *SPARC* transcription, with Enh2 showing the highest responsiveness to HDAC10 and p300 manipulation (Figure 3B). To investigate whether dCas9-p300 or dCas9-HDAC10 modulates *SPARC* transcription by altering its H3K27ac state, we narrowed our focus to Enh2. Cells co-expressing Enh2 gRNAs along with either dCas9-p300 or dCas9-HDAC10 were subjected to ChIP assays using antibodies to the FLAG epitope and H3K27ac. The recruitment of FLAG-tagged dCas9-fused proteins and the accumulation of H3K27ac at Enh2 and the promoter were then assessed. When co-expressing with Enh2 gRNAs, dCas9-p300 was significantly recruited to Enh2, but not to the promoter (Figure 3D); a significant increase in H3K27ac level was observed at Enh2, where dCas9-p300 was recruited, but not at the promoter (Figure 3E). In contrast, when dCas9-HDAC10 was recruited to Enh2 (Figure 3F), the H3K27ac level at Enh2 was discernibly decreased. Concurrently, the H3K27ac accumulation at the promoter remained relatively stable (Figure 3G). Cumulatively, these observations underline the notion that dCas9-HDAC10 and dCas9-p300 induce a bidirectional influence on the H3K27ac landscape at *SPARC* regulatory elements, thereby steering *SPARC* transcription.

H3K27ac shapes active promoters and enhancers by opening chromatin and recruiting transcription (co)factors to core promoters (58–61). Among these, BRD4 is noteworthy. It recognizes and binds to acetylated histones, and aids in gene transcription by recruiting other (co)factors to the target site and enhancing their functional activity (62–64). Of particular interest is its involvement in SPARC expression regulation; inhibition of BRD4 activity by small molecule inhibitors or RNA interference reduced SPARC expression (65). We wondered if BRD4 plays a role in HDAC10’s regulation of SPARC expression. To address this, cells were depleted of HDAC10 and treated with JQ1, a well-established BRD4 inhibitor that prevents BRD4 from binding to acetylated proteins. Surprisingly, while exposure to JQ1 did not significantly affect the basal expression of SPARC, it markedly blocked the SPARC upregulation arising from HDAC10 depletion (Figure 3H). These findings suggest that cells may maintain lower level of H3K27ac and accumulate less BRD4 at *SPARC* regulatory elements responsible for priming SPARC expression. Hence, BRD4 might not be essential for the priming of SPARC expression. However, upon HDAC10 depletion, the resulting increase in H3K27ac level may facilitate the recruitment of BRD4 to *SPARC* regulatory elements, such as Enh2, thereby promoting *SPARC* transcription.

To further investigate the potential involvement of H3K27ac in the recruitment of BRD4 to Enh2, cells co-expressing dCas9-p300 and Enh2 gRNAs were treated with JQ1. The *SPARC* expression level and BRD4 occupancy at Enh2 were evaluated. Indeed, the SPARC upregulation by the recruitment of dCas9-p300 to Enh2 requires the activity of BRD4 (Figure 3I). Consistent with the role of BRD4 in enhancer acetylation-dependent transactivation of SPARC, the BRD4 occupancy at Enh2 (but not at the promoter) was significantly enriched when dCas9-p300 was recruited to this region (Figure 3J). In line with this, when HDAC10 depletion induced BRD4 enrichment at Enh2 was attenuated due to BRD4 depletion (Figure 3K), the SPARC upregulation was diminished (Figure 3L); implying the induction of *SPARC* expression is likely due to increased H3K27ac level and BRD4 occupancy at *SPARC* regulatory elements.

### HDAC10 depletion represses melanoma cell growth by upregulating SPARC expression

**Figure 4.**
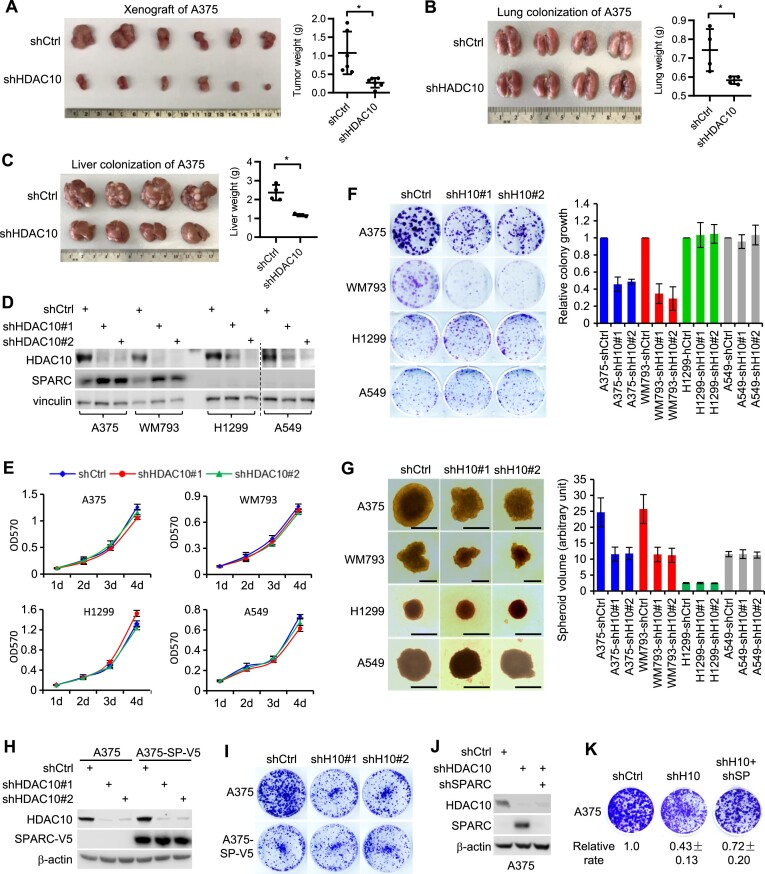
HDAC10 depletion represses melanoma cell growth by upregulating SPARC. (**A**) HDAC10-depleted and control A375 cells (5 × 10^5^ cells in 100 μl PBS) were subcutaneously injected into NSG mice. Xenograft growth was measured 20 days post-injection. (B, C) Lung and liver colonization by HDAC10-depleted and control A375 cells (1 × 10^6^ cells/injection) at 5 weeks after tail vein injection in NSG mice. The gross morphology of lung (left panel) and the weight of mice lung (right panel) are shown (**B**). The gross morphology of liver with tumor nodules (left panel) and weight of liver (left panel) are shown (**C**). (**D**) Following HDAC10 depletion, SPARC protein expression was evaluated. (**E**) Proliferative ability of HDAC10-depleted and control cells was assessed using MTT assay. (**F**) HDAC10-depleted and control cells were evaluated for their colony formation ability. Cells were seeded on 3.5-cm dishes or 6-well plates. For each, 500 (A375, WM793 and H1299) or 1000 cells (A549) were applied. Cells were stained with crystal violet (left panel) and relative colony growth of cells was quantified by ImageJ (right panel). (**G**) Spheroid growth of HDAC10-depleted and control melanoma and lung cancer cell lines was assessed. Representative spheroid morphology at day 6 are shown (left panel). Scale bar: 500 μm. Relative spheroid volumes are graphed as means ± SD of at least 10 spheroids (right). (**H**) A375 cells were depleted of HDAC10 and/or overexpressed with V5-tagged SPARC. Cell lysates were immunoblotted for HDAC10 and V5-tagged SPARC. (**I**) Colony growth of A375 cells depleted of HDAC10 and/or overexpressed with SPARC. (**J**) SPARC was further depleted in HDAC10-depleted or control A375 cells, and was confirmed by immunoblotting. (**K**) Colony growth of A375 cells depleted of SPARC and/or HDAC10. Relative growth rates are presented as means ± SD from three independent experiments.

Having uncovered HDAC10’s regulation in SPARC expression, we sought to explore its biological implications in cancer cells. SPARC regulates cell growth and proliferation in certain malignancies, including melanoma (53,66). Accordantly, we observed a marked inhibition in subcutaneous growth (Figure 4A) and a diminished capacity for lung and liver colonization (Figures 4B and C) in HDAC10-depleted A375 cells.

We next dissected the potential involvement of SPARC in HDAC10 depletion induced growth inhibition by MTT, colony formation and spheroid growth assays. It is worth noting that transient depletion of HDAC10 may induce severe cell cycle arrest and growth inhibition via downregulation of cyclin A expression (47). To eliminate this potential confounding factor, we established stable HDAC10-depleted cell lines by long-term culture (∼4 weeks), in which the acute cell cycle arrest and growth inhibition are restored. The melanoma (A375 and WM793) and lung cancer (H1299 and A549) cell lines were chosen for this study due to their differential SPARC protein expression levels. While HDAC10 depletion caused a significant SPARC upregulation in melanoma cells, SPARC expressions in lung cancer cells were too low to effectively gauge any change brought about by HDAC10 depletion (Figure 4D). Irrespective of the SPARC expression levels, HDAC10 depletion only marginally affected the proliferation of all tested cell lines under 2D culture condition, as determined by the MTT assay (Figure 4E). However, in melanoma cells characterized by high SPARC expression, HDAC10 depletion significantly repressed colony (Figure 4F) and spheroid (Figure 4G) growth. In contrast, in lung cancer cells where SPARC protein expression is low, HDAC10 depletion had minimal effect on colony or spheroid growth.

To further validate if SPARC is crucial for the growth suppression by HDAC10 depletion, the A375 cells were ectopically expressed with SPARC and/or depleted of HDAC10 (Figure 4H), then were examined for growth capacity (Figure 4I). Consistent with previous findings, ectopic expression of SPARC resulted in a reduced growth rate (20,28,29) and, consequently, overrode the inhibitory effect on colony growth induced by HDAC10 depletion. Conversely, when SPARC was depleted (Figure 4J), the colony growth repression was significantly blunted (Figure 4K). Collectively, HDAC10 depletion suppresses melanoma cell growth, at least in part, by an increase in SPARC expression.

### SPARC upregulation by HDAC10 depletion induces AMPK activation and autophagy

**Figure 5.**
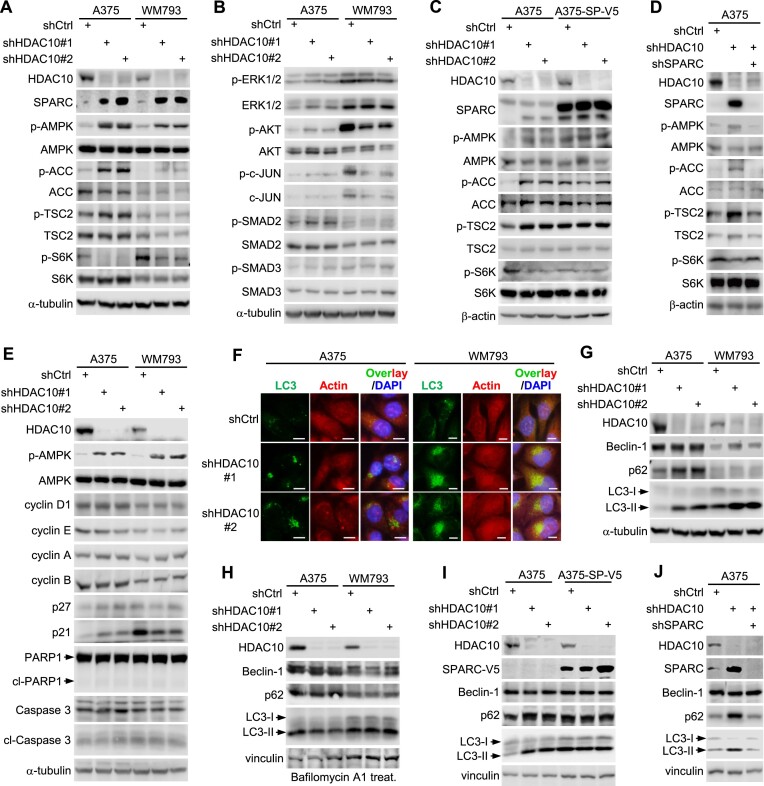
SPARC upregulation by HDAC10 depletion induces AMPK activation and autophagy. (**A, B**) A375 and WM793 cells were depleted of HDAC10. Cell lysates were blotted with antibodies to indicated proteins. (**C**) A375 cells were depleted of HDAC10 and/or overexpressed with SPARC. Cell lysates were blotted with antibodies to indicated proteins. (**D**) Lysates from A375 cells depleted of HDAC10 and/or SPARC were blotted with antibodies to indicated proteins. (**E**) Lysates from HDAC10-depleted or control A375 and WM793 cells were blotted with antibodies to indicated proteins. (**F**) HDAC10-depleted and control A375 and WM793 cells were stained for LC3 (green), actin (red), and nucleus (blue). (**G**) Lysates from HDAC10-depleted or control A375 and WM793 cells were blotted with antibodies to Beclin-1, p62 and LC3. (**H**) HDAC10-depleted and control A375 and WM793 cells were treated with 10 nM Bafilomycin A1 for 24 h. Lysates were blotted with antibodies to Beclin-1, p62 and LC3. (**I**) Lysates from A375 cells depleted of HDAC10 and/or overexpressed with V5-tagged SPARC were blotted with antibodies to indicated proteins. (**J**) Lysates from A375 cells depleted of HDAC10 and/or SPARC were blotted with antibodies to indicated proteins.

Next, we explored the mechanism underlying the growth repression related to the HDAC10–SPARC axis. SPARC influences multiple signaling pathways, including AMPK, MAPK and TGFβ/SMAD signaling, among others (17,19,67–73). These pathways play critical roles in cell growth, albeit in a cell-context-dependent manner. Therefore, the HDAC10-SPARC axis may regulate the colony and spheroid growth of melanoma cells by interfering with some of these pathways. To test this hypothesis, A375 and WM793 cells were depleted of HDAC10 and were examined for alterations in these signaling pathways by immunoblotting. Upon HDAC10 depletion, the phosphorylation of AMPK, indicative of its activation, was elevated in both cell lines (Figure 5A). Consistently, HDAC10 depletion markedly promoted the phosphorylation of acetyl-CoA carboxylase (ACC), a direct downstream target of AMPK (74), while attenuating the phosphorylation of S6K1, another downstream target of AMPK, likely through TSC2 phosphorylation (75–77). In contrast, the activities of other signalings either did not significantly change or showed alterations in only one of the cell lines, making it challenging to attribute the growth inhibition observed in these pathways (Figure 5B). Thus, it is likely that AMPK signaling activation, rather than changes in other pathways, primarily contributes to the growth suppression. We wondered whether the activation of AMPK results from HDAC10 depletion induced SPARC upregulation. A375 cells were ectopically expressed with SPARC and/or depleted of HDAC10, then were examined for the phosphorylation of AMPK and its downstream targets. Like HDAC10 depletion, overexpression of SPARC led to activation of AMPK and its downstream signaling (Figure 5C). Conversely, knockdown of SPARC attenuated the activation of AMPK signaling caused by HDAC10 depletion (Figure 5D); suggesting that HDAC10 depletion activates AMPK signaling in a manner dependent on SPARC upregulation.

AMPK activation can modulate cell growth and proliferation by affecting cell cycle progression, apoptosis, and/or autophagy, depending on the specific stimuli and cellular context (77–79). We examined the potential effects of the HDAC10-AMPK axis on the expression of key regulators of cell cycle progression (cyclins D1, E, A and B, as well as p21 and p27) and apoptosis (PARP1 and Caspase 3). Surprisingly, although HDAC10 depletion led to AMPK activation, it did not significantly change the expression patterns of these regulators in either cell line, or consistently in both cell lines (Figure 5E). Therefore, the cell growth inhibition caused by HDAC10 depletion did not result from cell cycle arrest or acute apoptosis; rather, it was likely due to the impact on autophagy. Supporting this hypothesis, stable HDAC10 depletion in melanoma cells caused remarkable increases in the LC3 autophagic puncta formation and accumulation, indicating an autophagy-like change (Figure 5F). HDAC10 depletion increased the conversion of LC3-1 to LC3-II and the level of p62 proteins (Figure 5G); in contrast, treatment with the lysosomal inhibitor Bafilomycin A1 mainly enhanced the accumulation of LC3-II (Figure 5H), suggesting an autophagy induction by HDAC10 depletion. SPARC was essential for HDAC10 depletion-induced AMPK activation, and thus likely influenced autophagy. To examine this, we manipulated the expression of SPARC in the A375 cells depleted of HDAC10 and then assessed the expression of LC3, p62, and Beclin-1. SPARC overexpression counteracted the effects of HDAC10 depletion and induced autophagy (Figure 5I). Conversely, SPARC depletion largely mitigated the autophagy elicited by HDAC10 depletion (Figure 5J). Collectively, these results indicate that the growth inhibition resulting from HDAC10 depletion is likely attributable to the AMPK activation and autophagy induction, via upregulation of SPARC.

### Expression of HDAC10 and SPARC is responsive to BRAF inhibition

Melanoma is the most aggressive form of skin cancer with a tendency to metastasize to other parts of the body if not detected and treated early. Approximately 50–60% of cutaneous melanomas harbor *BRAF* mutations, predominantly V600E. Targeted therapies that specifically inactivate mutated BRAF protein using BRAF inhibitor (BRAFi), such as vemurafenib (PLX4032), have yielded significant positive outcomes in patients with *BRAF* mutant melanoma. Yet, the emergence of resistance to these drugs remains a major challenge in long-term treatment.

**Figure 6.**
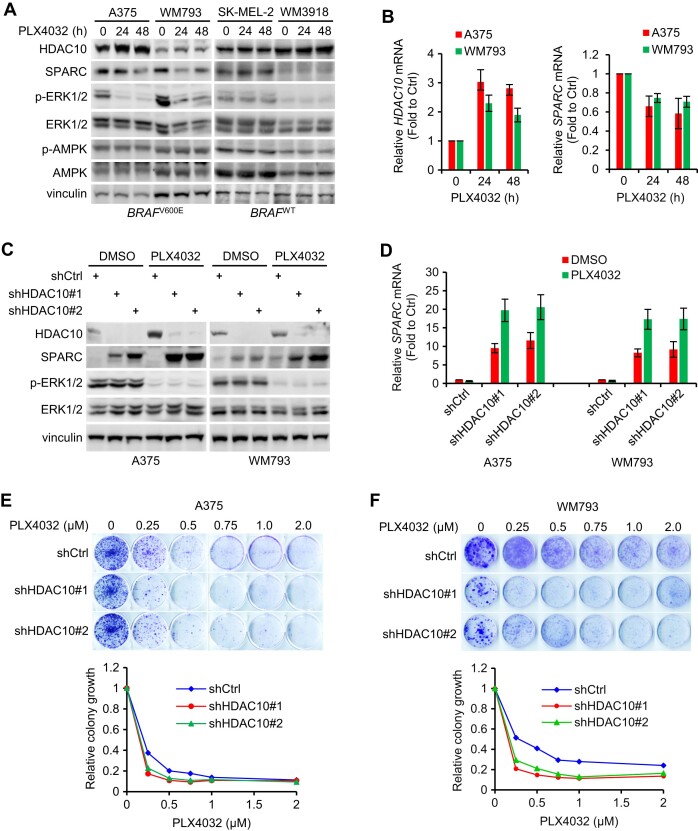
Expression of HDAC10 and SPARC is responsive to BRAF inhibition. (**A**) *BRAF* mutant (A375 and WM793) and WT (SK-MEL-2 and WM3918) melanoma cells were treated with 1 μM PLX4032 for 24 h or 48 h. Cell lysates were blotted with antibodies to indicated proteins. (**B**) The relative mRNA levels of *HDAC10* and *SPARC* of A375 and WM793 cells were quantified by qPCR. (C, D) HDAC10-depleted or control cells underwent PLX4032 treatment. Lysates were blotted with antibodies to indicated proteins (**C**). The relative *SPARC* mRNA levels were quantified by qPCR (**D**). (**E, F**) HDAC10-depleted and control cells were seeded on 6-well plates (2000 cells for each well) and treated with indicated doses of PLX4032. Cells were stained with crystal violet (upper panel). Relative colony growth was quantified by ImageJ (lower panel).

HDAC10 and SPARC are implicated in chemotherapy response across different types of cancer (10,37,80–83), but their roles in BRAFi resistance in melanoma are unexplored. Notably, the expression dynamics of both HDAC10 and SPARC are influenced by the BRAF activity state in *BRAF* mutant melanoma cells (A375 and WM793) (Figures 6A and B). While it had minimal impact on AMPK expression and activation, PLX4032 treatment, evidenced by decreased phospho-ERK1/2 level, resulted in increased HDAC10 at both protein and mRNA levels, subsequently reducing SPARC levels. In contrast, treatment with PLX4032 didn’t significantly change the expression patterns of HDAC10 and SPARC in *BRAF* WT melanoma cells (SK-MEL-2 and WM3918). Further, when exposed to PLX4032, HDAC10-depleted melanoma cells exhibited more pronounced inductions of both SPARC protein and mRNA (Figures 6C and D). These findings not only reiterate HDAC10’s suppressive impact on SPARC expression but also spotlight its potential role in modulating SPARC response to PLX4032. Importantly, HDAC10 knockdown appears to confer an additional augmentation of sensitivity to PLX4032 in A375 and WM793 cells (Figures 6E and F). However, since both cell lines are sensitive to PLX4032, it remains to be conclusively determined whether HDAC10 depletion inherently alters melanoma cell susceptibility to BRAFi.

### HDAC10 depletion overcomes BRAF inhibitor resistance partially through upregulating SPARC expression

**Figure 7.**
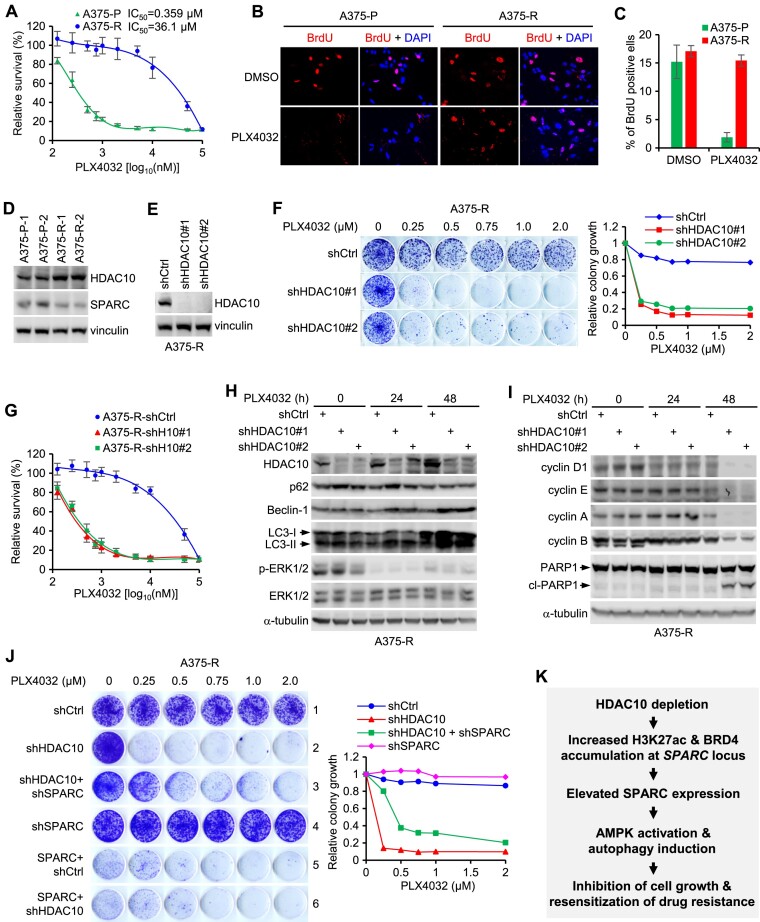
HDAC10 depletion overcomes BRAF inhibitor resistance partially through upregulating SPARC. (**A**) Parental (A375-P) and PLX4032-resistant (A375-R) A375 cells underwent gradient PLX4032 treatments for 72 h. Cell viability was ascertained using the CCK8 assay, with data displayed as mean ± SD over three repetitions. (B, C) Cells were treated with PLX4032 (2 μM) or the DMSO control for 24 h, and subjected to BrdU incorporation assay. Cells were stained for BrdU (red) and nucleus (blue). Representative BrdU staining images are provided (**B**), and BrdU incorporation rates are graphed as mean ± SD from three experiments (**C**). For each, at least 300 cells are counted. (**D**) Lysates from A375-P and A375-R cells were blotted for HDAC10 and SPARC. (**E**) Lysates from HDAC10-depleted and control A375-R cells were blotted for HDAC10 and vinculin. (**F**) HDAC10-depleted and control A375-R cells were seeded on 6-well plated, and treated with indicated doses of PLX4032 for 10 days. Cells were stained with crystal violet (left panel), and relative colony growth of cells was graphed (right panel). (**G**) HDAC10-depleted and control cells were treated with gradient doses of PLX4032 for 72 h. Relative cell viability was evaluated by CCK8 assay. (**H, I**) HDAC10-depleted and control A375-R cells were treated with PLX4032 for 24 h or 48 h. Cell lysates were blotted with antibodies to indicated proteins. (**J**) A375-R cells were depleted of HDAC10, and/or were depleted of or overexpressed with SPARC. Cells were then treated with gradient doses of PLX4032 for 10 days. Cells were stained with crystal violet (upper panel) and relative colony growth of cells was graphed (lower panel). (**K**) Schematic diagram of the proposed role of HDAC10 in regulating SPARC expression, melanoma cell growth and drug resistance.

We next developed a PLX4032-resistant cell line (A375-R) through chronic *in vitro* exposure to PLX4032. A375-R cells exhibited a markedly higher inhibitory potency (IC_50_) to PLX4032 compared to the parental A375 cells (A375-P) (Figure 7A). While A375-P cell proliferation was effectively inhibited by PLX4032, A375-R cells retained their proliferative vigor in its presence (Figures 7B and C).

Intriguingly, we observed an upregulation of HDAC10 and a concurrent downregulation of SPARC in A375-R cells in comparison to A375-P cells (Figure 7D). This finding, in conjunction with those presented in Figure 6, suggests that the shifts in the expression levels of HDAC10 and SPARC by BRAF inhibition are not transient or stochastic responses. Instead, they exhibit relative stability, which could have significant and enduring implications for melanoma treatment strategies involving BRAFi. To gain insight into the potential influence of HDAC10 on BRAFi resistance, A375-R cells were depleted of HDAC10 (Figure 7E), subsequently exposed to various concentrations of PLX4032, and were evaluated for viability using colony formation and CCK8 assays. Depletion of HDAC10 resulted in a noteworthy reduction in colony growth (Figure 7F) and, consistently, a substantial restoration of sensitivity to PLX4032 (Figure 7G). In concordance with these findings, HDAC10 depletion led to an augmentation of cellular autophagy upon PLX4032 treatment, as indicated by increased expression of autophagic markers such as LC3, p62 and Beclin-1 (Figure 7H). Since the vulnerability to PLX4032 was observed in both colony formation and CCK8 assays, HDAC10 depletion may induce acute cell cycle arrest, potentially accompanied by cell death, in addition to autophagic response. Supporting this hypothesis, exposure to PLX4032 for 48 h significantly downregulated the expression of cyclins while upregulating the expression of cleaved PARP1 in HDAC10-depleted cells (Figure 7I). Thus, HDAC10 depletion may influence melanoma cell fate in diverse ways depending on the cellular context. In situations where cellular damage or stress is severe and the cell's ability to adapt or recover is compromised without HDAC10, besides autophagy, acute cell cycle arrest and apoptosis may occur.

Finally, we asked if SPARC has a role in HDAC10-mediated regulation of PLX4032 resistance in A375-R cells. To this end, HDAC10-depleted or control A375-R cells were further depleted of or overexpressed with SPARC, then were evaluated for PLX4032 response. As expected, HDAC10 depletion rendered A375-R cells vulnerable to PLX4032 (Figure 7J, left panel, rows 1 and 2). Intriguingly, while the sole depletion of SPARC (SPARC was depleted in A375-R cells) had little effect on, the double depletion of SPARC (SPARC was depleted in HDAC10-depleted A375-R cells) resulted in a partial yet noticeable restoration of, sensitivity to the PLX4032 (Figure 7J, left panel, rows 3 and 4). These results imply that HDAC10 likely involves PLX4032 response regulation through multiple pathways, besides SPARC signaling. Additionally, these findings suggest that it is SPARC upregulation induced by HDAC10 depletion, but not the intrinsic SPARC expression, that plays a role in regulating PLX4032 response in A375-R cells. Supporting this idea, overexpression of SPARC markedly reduced colony growth and PLX4032 resistance in A375-R cells, regardless of the HDAC10 status (Figure 7J, left panel, rows 5 and 6).

In summary, our studies reveal that HDAC10 plays a critical role in the regulation of SPARC expression in the melanoma cells. Knockdown or inhibition of HDAC10 leads to an increase in H3K27ac levels and promotes the accumulation of BRD4 at *SPARC* locus, consequently resulting in the upregulation of SPARC expression. Depletion of HDAC10 and the subsequent upregulation of SPARC activate AMPK signaling and induce autophagy, ultimately suppressing melanoma cell growth and attenuating resistance to BRAF inhibitors (Figure 7K).

## Discussion

HDAC10 belongs to the class IIb HDAC family, which was initially identified based on its sequence similarity to other class II HDACs (84–87). Particularly, it shares the highest structural resemblance with HDAC6, the other member of class IIb HDACs. Despite their structural similarity, they have distinct functions and subcellular localizations. HDAC6 is primarily cytoplasmic, mainly deacetylating cytosolic targets and guiding related processes (88,89). HDAC10, on the other hand, is potentially more versatile with its presence both in the cytoplasm and the nucleus. This dual localization hints at a broader role for HDAC10, potentially in nuclear processes that HDAC6 might not partake in. HDAC10’s involvement in critical cellular processes, such as cell proliferation, apoptosis, and autophagy, highlight its potential impact on cancer progression (80,90). This has naturally led to a growing interest in targeting HDAC10 for cancer therapy, culminating in the development of selective HDAC10 inhibitors (55,91–93). These pharmacological agents are instrumental in deciphering HDAC10’s biological functions, and offer promising avenues for novel cancer treatments.

Our study delves deeper into HDAC10’s specific regulatory influence on SPARC expression; its depletion sharply elevates SPARC level, while its overexpression does the opposite. The interplay between HDAC10 and histone modifications was another focal point of our research. We established a correlation between HDAC10 and H3K27ac, a modification often associated with gene activation, at *SPARC* regulatory elements. Harnessing the precision of a dCas9-mediated CRISPR approach, our study elucidated the competition between HDAC10 and p300/CBP, two crucial regulators of H3K27ac, at *SPARC* Enh2. These findings shed light on the mechanisms governing SPARC expression and highlight the potential of targeting HDAC10 in therapeutic strategies.

Although HDAC10 was identified over two decades ago (84–87,94), the physiological function of HDAC10 as a lysine deacetylase remains ambiguous and controversial. In early studies, using either purified core histones or synthetic histone H4 peptide substrates, anti-FLAG immunoprecipitates derived from FLAG-tagged HDAC10 expressing cells exhibited histone deacetylase activities (84–87). Further, these same groups show that HDAC10 possesses transcriptional repression activity when targeted to promoters as a Gal4-fusion protein, though there were disagreements about whether repression by HDAC10 requires its intrinsic histone deacetylase activity. Later, using purified recombinant HDAC10, the enzymatic activity of HDAC10 could not be determined with a wide range of fluorophore-conjugated substrates (95,96). These data raised the intriguing question of whether HDAC10 truly possesses lysine deacetylase activity or has a very limited number of substrates that may involve non-lysine and non-proteins. The answer came with the breakthrough discovery that HDAC10 is a robust polyamine deacetylase, with optimal catalytic activity and specificity for the hydrolysis of N^8^-acetylspermidine (97). While these recent results clearly demonstrate that recombinant HDAC10 has negligible or low lysine deacetylase activity *in vitro*, some reports support earlier findings that HDAC10 catalyzes protein deacetylations. For instance, HDAC10 catalyzes the deacetylation of non-histone proteins including Hsc70/Hsp70 (98), EWSR1 (99), YAP1 (100), MSH2 (94) and hSSB1 (101). Conceivably, HDAC10 may associate with other HDACs to indirectly deacetylate proteins in living cells. This possibility is reinforced by two early reports that HDAC10 interacts with HDAC2 and HDAC3 (84,87). Also, HDAC10 has been shown to repress the promoter of CXCL10 by recruitment of EZH2 (102), OsHKT2 by interaction with OsPRR73 (103), DUB3 by complexing with NCOR2 (104), and MMP2/9 by inhibiting RNA polymerase II (105).

Our observation that HDAC10 modulates the H3K27ac state at *SPARC* locus and represses its expression does not endorse that HDAC10 possesses intrinsic histone deacetylase activity. Rather, it fits well with the hypothesis that HDAC10 interacts with other HDACs or acetylation-modifying enzymes to indirectly regulate histone/protein modifications in cells. Alternatively, though non-mutually exclusive, with its robust polyamine deacetylase activity towards N^8^-acetylspermidine, it is possible that HDAC10 depletion leads to an increase in acetylspermidine level, resulting in deregulated polyamine metabolism. This deregulation can potentially impact the activities of p300/CBP and other histone/protein acetyltransferases and, consequently, the acetylation state of histone and non-histone proteins, by affecting the availability of polyamine-derived acetyl groups for acetyltransferase enzymes (106,107). Considering the widespread nature of (acetyl)spermidine, it is conceivable that HDAC10’s absence would influence the overall H3K27ac level in cells. Yet, our work indicates histone deacetylation at specific genes, such as *SPARC*. We hypothesize that the observed rise in H3K27ac level at *SPARC* regulatory elements upon HDAC10 depletion is not due to spermidine's deacetylation. Other alternative mechanisms could be at play. First, HDAC10 may counteract or compete with p300/CBP and/or other HDACs at specific sites, thereby affecting the access of HATs and/or HDACs to the local histones surrounding the gene promoter or enhancers and influencing the acetylation state. In this scenario, HDAC10 may work similarly to HDAC8 which modulates p300 function, and increases H3K27ac and chromatin accessibility at Jun-transcriptional sites in melanoma cells (108). Second, HDAC10 could interact with transcription (co)factors, in turn, modulating the accessibility of p300 to histone H3. In addition to HDAC2 and SMRT, several (co)factors, such as TP53INP1 and SOX5, are known to impact *SPARC* transcription and may work in tandem with HDAC10 (109–114). The precise mechanism by which HDAC10 influences the H3K27ac state at *SPARC* locus warrants further exploration.

SPARC is involved in various aspects of development and growth of melanoma. It exhibits both tumor-promoting and tumor-suppressing effects, depending on the context and stage of melanoma progression (10,11). Our work indicates that the growth inhibition of melanoma cells is closely linked to SPARC upregulation following stable HDAC10 depletion. The transient depletion of HDAC10 may lead to severe mitotic catastrophe and cell cycle arrest in 2D culture in some melanoma lines. After bypassing a proliferative crisis, the surviving HDAC10-depleted cells that restore a similar proliferative ability in 2D monolayer culture retain an upregulation of SPARC. In this regard, the acute cell death and cell cycle arrest occurring at an early stage upon HDAC10 depletion is unlikely due to the upregulation of SPARC. In support of this perspective, the expression levels of cell cycle and apoptosis regulators remain relatively stable in these HDAC10-depleted cells, compared to that in the control cells. Instead, as time progresses post-HDAC10 depletion, the inhibitory effect on cell growth, attributed to the SPARC upsurge, seems to result from autophagy.

Our study further deciphers the underlying relationship between HDAC10, SPARC, and AMPK; the HDAC10 depletion leads to the upregulation of SPARC, in turn, heightening the phosphorylation of AMPK. Corroboratively, overexpressing SPARC elevates AMPK phosphorylation, effectively overshadowing the influence of HDAC10 depletion. Inversely, SPARC depletion dampens the activation of AMPK upon HDAC10 depletion. In essence, the activation of AMPK signaling upon HDAC10 depletion hinges on the upregulation of SPARC.

SPARC’s multifaceted roles encompass aspects such as cell adhesion, migration, proliferation, and survival. In certain cell types, SPARC maintains an intracellular presence, implying it may have unexplored intracellular functions (22,25,115–119). SPARC’s roles in mediating AMPK activation, facilitating glucose metabolism, and interacting with metabolic pathways are well-documented (67,68,120). Moreover, SPARC-induced AMPK activation has been linked to autophagy (121). In our study, AMPK activation, triggered by HDAC10 depletion and concurrent SPARC upregulation, was found to potently induce autophagy, as reflected in the marked conversion of LC3-I to LC3-II. The depletion of HDAC10 has a minimal effect on apoptosis and cell cycle progression in melanoma cells. Instead, the suppression of melanoma cell growth appears to be primarily attributed to autophagy, a consequence of AMPK activation.

Our study also demonstrates that the expression levels of both HDAC10 and SPARC respond to the activity state of mutated BRAF in melanoma cells. Furthermore, our findings unveil a previously unrecognized role of the HDAC10-SPARC axis in modulating BRAFi response in *BRAF* mutant melanoma cells; HDAC10 depletion significantly re-sensitizes PLX4032-resistant melanoma cells to PLX4032 treatment, while SPARC depletion partially reverses this effect. For the first time, our findings underscore the significance of HDAC10 and SPARC in BRAFi resistance. These results also suggest that HDAC10 may influence BRAFi response through multiple downstream targets beyond SPARC. Further investigation is warranted to fully elucidate the underlying mechanisms.

In summary, our study marks a significant stride in understanding how HDAC10 controls *SPARC* expression, specifically through modulating H3K27ac state at its regulatory elements. Our work also establishes the HDAC10-SPARC axis as a critical regulator in melanoma cell growth and BRAFi resistance, offering insight into potential therapeutic targets for melanoma and related malignancies.

## Supplementary Material

zcae018_Supplemental_Files

## Data Availability

The authors confirm that the data supporting the findings of this study are available within the article and its supplementary materials. Raw data and derived data supporting the findings of this study are available from the authors on request.
