# Supplementary material for: HDAC10 inhibition represses melanoma cell growth and BRAF inhibitor resistance via upregulating SPARC expression
Source: NAR Cancer. 2024 Apr 22;6(2):zcae018. doi: 10.1093/narcan/zcae018 (PMC11034028; doi:10.1093/narcan/zcae018)
Supplement: zcae018_Supplemental_Files [file zcae018_supplemental_files.zip › Figure S1.pdf]

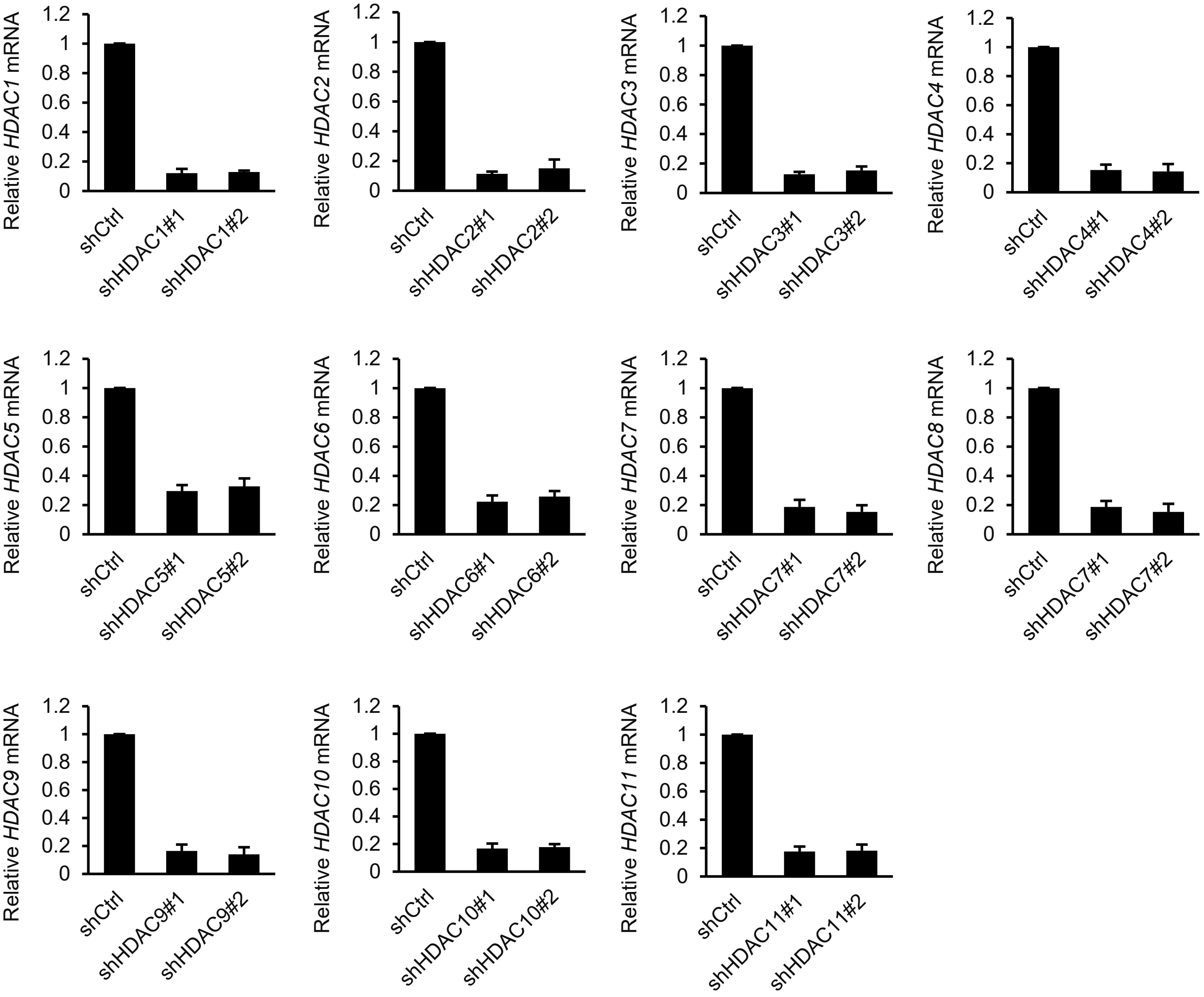

Figure S1. Evaluation of the knockdown of HDAC1–11 in A375 melanoma cells by qPCR.

A375 melanoma cells were infected with lentiviruses harboring shRNA targeting individual HDAC member (HDAC1–11) and control. Relative mRNA level of each HDAC was determined by qPCR.
