# Supplementary material for: HDAC10 inhibition represses melanoma cell growth and BRAF inhibitor resistance via upregulating SPARC expression
Source: NAR Cancer. 2024 Apr 22;6(2):zcae018. doi: 10.1093/narcan/zcae018 (PMC11034028; doi:10.1093/narcan/zcae018)
Supplement: zcae018_Supplemental_Files [file zcae018_supplemental_files.zip › Figure S2.pdf]

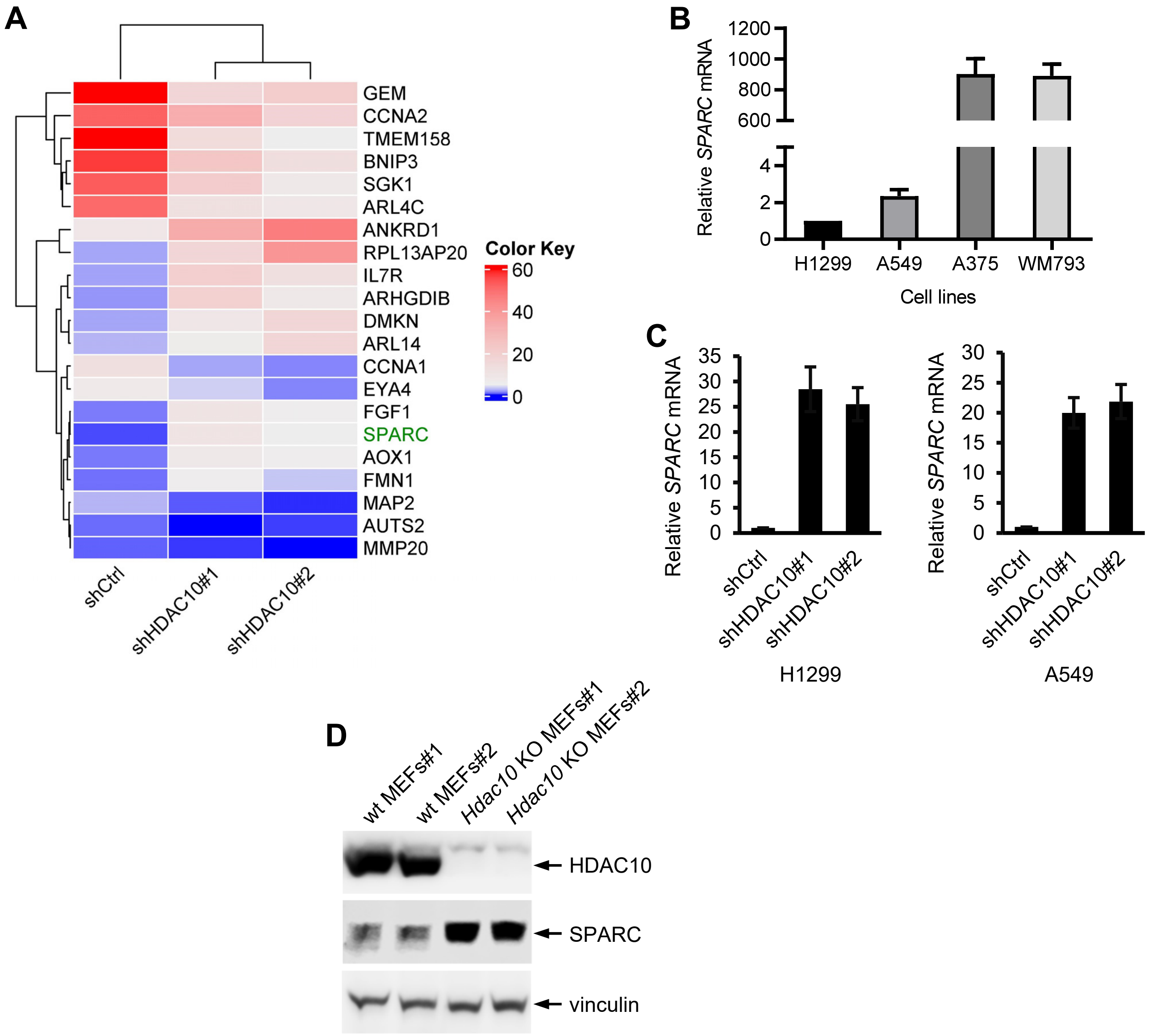

Figure S2. HDAC10 depletion or deletion upregulates the levels of *SPARC* mRNA or protein in lung cancer cells and mouse embryonic fibroblasts (MEFs), respectively.

(A) The top differential expression genes HDAC10 depletion in H1299 lung cancer cells by RNA-seq.

(B) Comparison of relative mRNA levels of *SPARC* between lung cancer (H1299 and A549) and melanoma (A375 and WM793) cell lines.

(C) HDAC10 depletion upregulates the mRNA level of *SPARC* in lung cancer cell lines.

(D) Compared to the wt MEFs, *Hdac10* KO MEFs display higher expression levels of SPARC protein.
