# Supplementary material for: HDAC10 inhibition represses melanoma cell growth and BRAF inhibitor resistance via upregulating SPARC expression
Source: NAR Cancer. 2024 Apr 22;6(2):zcae018. doi: 10.1093/narcan/zcae018 (PMC11034028; doi:10.1093/narcan/zcae018)
Supplement: zcae018_Supplemental_Files [file zcae018_supplemental_files.zip › Table S1_12Mar2024.pdf]

Table S1. shRNAs and oligonucleotides used in this paper

shRNAs for gene knockdown

shRNAs	Target sequences (from 5' to 3')	Sources	Identifier
HDAC1-shRNA#1	CGGTTAGGTTGCTTCAATCTA	Sigma	TRCN0000195467
HDAC1-shRNA#2	CCTAATGAGCTTCCATACAAT	Sigma	TRCN0000195103
HDAC2-shRNA#1	GCAAATACTATGCTGTCAATT	Sigma	TRCN0000004823
HDAC2-shRNA#2	CAGACTGATATGGCTGTTAAT	Sigma	TRCN0000195198
HDAC3-shRNA#1	CAAGAGTCTTAATGCCTTCAA	Sigma	TRCN0000194993
HDAC3-shRNA#2	CCTTCCACAAATACGGAAATT	Sigma	TRCN0000004825
HDAC4-shRNA#1	GCCAAAGATGACTTCCCTCTT	Sigma	TRCN0000004832
HDAC4-shRNA#2	GCCAAAGATGACTTCCCTCTT	Sigma	TRCN0000314665
HDAC5-shRNA#1	GCTAGAGAAAGTCATCGAGAT	Sigma	TRCN0000004835
HDAC5-shRNA#2	GCTCAAGAATGGATTTGCCAT	Sigma	TRCN0000004836
HDAC6-shRNA#1	CGGTAATGGAACTCAGCACAT	Sigma	TRCN0000004842
HDAC6-shRNA#2	CATCCCATCCTGAATATCCTT	Sigma	TRCN0000314976
HDAC7-shRNA#1	GATCCGGGTGCACAGTAAATA	Sigma	TRCN0000255687
HDAC7-shRNA#2	CAAGTAGTTGGAACCAGAGAA	Sigma	TRCN0000195442
HDAC8-shRNA#1	GCGTATTCTCTACGTGGATTT	Sigma	TRCN0000350469
HDAC8-shRNA#2	GCGTATTCTCTACGTGGATTT	Sigma	TRCN0000004851
HDAC9-shRNA#1	CAAACTGCTTTCGAAATCTAT	Sigma	TRCN0000195059
HDAC9-shRNA#2	GAGCAGTTAATAGGCTTTAAA	Sigma	TRCN0000196384
HDAC10-shRNA#1	CCTGTACCTCTTAGATGGGAT	Sigma	TRCN0000004860
HDAC10-shRNA#2	GTGTTCAACAACGTGGCCATA	Sigma	TRCN0000004861
HDAC11-shRNA#1	CCCGACGTGGTGGTATACAAT	Sigma	TRCN0000199149
HDAC11-shRNA#2	GCGCTATCTTAATGAGCTCAA	Sigma	TRCN0000330863
SPARC-shRNA	CGGTTGTTCTTTCCTCACATT	Sigma	TRCN0000008709
p300-shRNA	CAATTCCGAGACATCTTGAGA	Sigma	TRCN0000009883
BRD4-shRNA	CCTGGAGATGACATAGTCTTA	Sigma	TRCN0000021427
Control-shRNA	GCAAGCTGACCCTGAAGTTCAT	Addgene	#30323

Primers for RT-qPCR

Primers	Sequences (from 5' to 3')	Sources	Identifier
HDAC1-RT-F	CGAATCCGCATGACTCATAA	IDT	This paper
HDAC1-RT-R	CATCTCCTCAGCATTGGCTT	IDT	This paper
HDAC2-RT-F	ATGGCGTACAGTCAAGGAGG	IDT	This paper
HDAC2-RT-R	ATGAGGCTTCATGGGATGAC	IDT	This paper
HDAC3-RT-F	GCAAGGCTTCACCAAGAGTC	IDT	This paper
HDAC3-RT-R	CTGTGTAACGCGAGCAGAAC	IDT	This paper
HDAC4-RT-F	CGTGGAAATTTTGAGCCATT	IDT	This paper
HDAC4-RT-R	CTGGTCTCGGCCAGAAAGT	IDT	This paper
HDAC5-RT-F	GGGAACCATCCTTGGAAATC	IDT	This paper
HDAC5-RT-R	GAACTGGGCATGGCTCTTG	IDT	This paper
HDAC6-RT-F	CCGGAGGGTCCTTATCGTAG	IDT	This paper
HDAC6-RT-R	GCGGTGGATGGAGAAATAGA	IDT	This paper
HDAC7-RT-F	CTGCATTGGAGGAATGAAGCT	IDT	This paper
HDAC7-RT-R	CTGGCACAGCGGATGTTTG	IDT	This paper
HDAC8-RT-F	ATACTTGACCGGGGTCATCC	IDT	This paper
HDAC8-RT-R	GCGTGATTTCCAGCACATAA	IDT	This paper
HDAC9-RT-F	GTACAGAAGTAAAGCAGAAG	IDT	This paper
HDAC9-RT-R	AGAGCTTTGATCCAATGATG	IDT	This paper

HDAC10-RT-F	CTCGGCTTCACTGTCAACCT	IDT	This paper
HDAC10-RT-R	GTCAAATCCTGCCGAGACCA	IDT	This paper
HDAC11-RT-F	GCACACGAGGCGCTATCTTA	IDT	This paper
HDAC11-RT-R	AAGGAAGTTGGGGAGGAAGA	IDT	This paper
SPARC-RT-F	ACATAAGCCCAGTTCATCACCA	IDT	This paper
SPARC-RT-R	ACAACCGATTCACCAACTCCA	IDT	This paper
GAPDH-RT-F	AAGGTGAAGGTCGGAGTCAA	IDT	This paper
GAPDH-RT-R	AATGAAGGGGTCATTGATGG	IDT	This paper

Oligonucleotides used for generation of plentiCRISPR-gRNA targeting *HDAC10*

**Oligonucleotides**	**Sequences (from 5' to 3')**	**Sources**	**Identifier**
HDAC10-gRNA-F	CACCGGACGCTCGATCTCGCACTCG	IDT	This paper
HDAC10-gRNA-R	AAACCGAGTGCGAGATCGAGCGTCC	IDT	This paper

Oligonucleotides used for generation of dCas9/CRISPR-gRNAs targeting *SPARC* loci

**Oligonucleotides**	**Sequences (from 5' to 3')**	**Sources**	**Identifier**
Pro-gRNA1-F	CACCGGATCTGCCCTGGGCTGACCA	IDT	This paper
Pro-gRNA1-R	AAACTGGTCAGCCCAGGGCAGATCC	IDT	This paper
Pro-gRNA2-F	CACCGGTCTGTCCCTTGGTCAGCCC	IDT	This paper
Pro-gRNA2-R	AAACGGGCTGACCAAGGGACAGACC	IDT	This paper
Pro-gRNA3-F	CACCGGAATGATGCAGTCTGTCCCT	IDT	This paper
Pro-gRNA3-R	AAACAGGGACAGACTGCATCATTCC	IDT	This paper
Pro-gRNA4-F	CACCGGACTGCATCATTCAGTCCAC	IDT	This paper
Pro-gRNA4-R	AAACGTGGACTGAATGATGCAGTCC	IDT	This paper
Pro-gRNA5-F	CACCGGAGATGTAACATTTGCCCTG	IDT	This paper
Pro-gRNA5-R	AAACCAGGGCAAATGTTACATCTCC	IDT	This paper
Enh1-gRNA1-F	CACCGGCTCAGTCTGGTGCTGATAA	IDT	This paper
Enh1-gRNA1-R	AAACTTATCAGCACCAGACTGAGCC	IDT	This paper
Enh1-gRNA2-F	CACCGAAGGATATAGCTGCTCAGTC	IDT	This paper
Enh1-gRNA2-R	AAACGACTGAGCAGCTATATCCTTC	IDT	This paper
Enh1-gRNA3-F	CACCGCAGTCTGGTGCTGATAATGG	IDT	This paper
Enh1-gRNA3-R	AAACCCATTATCAGCACCAGACTGC	IDT	This paper
Enh1-gRNA4-F	CACCGTAATGGTGGTAGTGATCAAA	IDT	This paper
Enh1-gRNA4-R	AAACTTTGATCACTACCACCATTAC	IDT	This paper
Enh1-gRNA5-F	CACCGCTATATCCTTTTATTAATCA	IDT	This paper
Enh1-gRNA5-R	AAACTGATTAATAAAAGGATATAGC	IDT	This paper
Enh2-gRNA1-F	CACCGGCTCCACACCCAGCCTTGGG	IDT	This paper
Enh2-gRNA1-R	AAACCCCAAGGCTGGGTGTGGAGCC	IDT	This paper
Enh2-gRNA2-F	CACCGGAGGAAAGGATATTTAAAGC	IDT	This paper
Enh2-gRNA2-R	AAACGCTTTAAATATCCTTTCCTCC	IDT	This paper
Enh2-gRNA3-F	CACCGGCAGAGCTAGGGGTAGACCT	IDT	This paper
Enh2-gRNA3-R	AAACAGGTCTACCCCTAGCTCTGCC	IDT	This paper
Enh2-gRNA4-F	CACCGGGCAGAGCTAGGGGTAGACC	IDT	This paper
Enh2-gRNA4-R	AAACGGTCTACCCCTAGCTCTGCCC	IDT	This paper
Enh2-gRNA5-F	CACCGGCAGTCAGTGGCAGAGCTAG	IDT	This paper
Enh2-gRNA5-R	AAACCTAGCTCTGCCACTGACTGCC	IDT	This paper
Enh3-gRNA1-F	CACCGGAGATCGACTAGGACTCCAT	IDT	This paper
Enh3-gRNA1-R	AAACATGGAGTCCTAGTCGATCTCC	IDT	This paper
Enh3-gRNA2-F	CACCGATGTCGCTGTACTAAACCTT	IDT	This paper
Enh3-gRNA2-R	AAACAAGGTTTAGTACAGCGACATC	IDT	This paper

Enh3-gRNA3-F	CACCGTACAGTTGTACTCACACCTA	IDT	This paper
Enh3-gRNA3-R	AAACTAGGTGTGAGTACAACTGTAC	IDT	This paper
Enh3-gRNA4-F	CACCGACAACTGTATGCTGAGCCAA	IDT	This paper
Enh3-gRNA4-R	AAACTTGGCTCAGCATACAGTTGTC	IDT	This paper

Primers for chromatin immunoprecipitation (ChIP)-qPCR

**Primers**	**Sequences (from 5' to 3')**	**Sources**	**Identifier**
Control-ChIP-F	ATGTACTGGGGGTGTACGGA	IDT	This paper
Control-ChIP-R	CCAGCCACAGCCTTAGAACT	IDT	This paper
Pro-ChIP-F	TGAGTCGGTTTAGGCAGCAG	IDT	This paper
Pro-ChIP-R	TCCTCCTCTTTTTGCGGTGG	IDT	This paper
Enh1-ChIP-F	CCCAAGACTAGTGTCCAGCC	IDT	This paper
Enh1-ChIP-R	CGGCGAAGAAAGGTAGTGGA	IDT	This paper
Enh2-ChIP-F	CACCCACTGGCTCAGCTAAA	IDT	This paper
Enh2-ChIP-R	CAGCCTGTAATAGGCGAGGG	IDT	This paper
Enh3-ChIP-F	AGATGGGGGAACCAAGGTCT	IDT	This paper
Enh3-ChIP-R	GCCAGGGGGTCTTGTTCTAC	IDT	This paper
